# Scapulothoracic Dissociation Following a Fall From Standing Height: A Rare and Atypical Presentation of Severe Shoulder Girdle Injury

**DOI:** 10.7759/cureus.80652

**Published:** 2025-03-16

**Authors:** Dimitrios Giotis, Christos Konstantinidis, Christos Koukos, Dimitrios Vardakas, Sotiris Plakoutsis

**Affiliations:** 1 Department of Orthopaedics, General Hospital of Ioannina "G. Hatzikosta", Ioannina, GRC; 2 Orthopaedics, Sports Trauma and Pain Institute, Thessaloniki, GRC

**Keywords:** atypical presentation, hemodynamic stabilization, injury mechanism, musculature, scapulothoracic dissociation

## Abstract

Scapulothoracic dissociation (SD) is a rare and severe injury, typically associated with high-energy trauma. It is characterized by scapular displacement, often leading to neurovascular compromise. SD resulting from low-energy mechanisms, such as a fall from standing height, is exceptionally rare. We present the case of a 93-year-old male who sustained SD after a fall from standing height. He was hemodynamically unstable upon arrival, with progressive neurological deficits in the right upper limb. Computed tomography (CT) revealed an isolated right scapular glenoid fracture with lateral scapular displacement and a large hematoma, while CT angiography ruled out major vascular injury. Conservative management included hemodynamic stabilization and immobilization with a Velpeau-type splint. One month post-injury, neurological function in the affected upper extremity had significantly improved. By five months, the patient had regained nearly full, painless shoulder range of motion without neurological deficits. Although SD is classically associated with high-energy trauma, this case highlights its occurrence following minor trauma in elderly patients. Clinicians should maintain a high index of suspicion for SD in atypical presentations to facilitate early diagnosis and optimize treatment.

## Introduction

Scapulothoracic dissociation (SD) is a rare but severe injury characterized by the traumatic separation of the scapula from the thoracic wall, frequently leading to neurovascular compromise [1]. First described by Oreck et al. in 1984, SD is most commonly associated with high-energy trauma, including motorcycle crashes, pedestrian-versus-vehicle collisions, and significant falls, which can cause a total disruption of the scapulothoracic articulation and result in the lateral displacement of the scapula [1,2].

The injury mechanism of SD typically involves violent traction forces applied to the upper extremity, causing disruption of the surrounding soft tissues, musculature, and ligamentous attachments [3]. In most cases, these forces lead to accompanying fractures, dislocations of the acromioclavicular or sternoclavicular joints, and damage to the underlying neurovascular structures [3]. Clinically, patients often present with massive shoulder swelling due to edema and hematoma, gross instability of the joint, absent distal pulses due to vascular injury, and significant neurologic deficits, with a high incidence of brachial plexus involvement [1-3].

The management of SD is complex and usually demands a multidisciplinary approach. Initial treatment prioritizes stabilization, pain control, and immobilization, while vascular injuries require urgent surgical intervention [4]. Neurogenic injuries may lead to poor functional outcomes, potentially resulting in flail extremity and, in severe cases, amputation [3,4]. Despite advances in early diagnosis and surgical management, morbidity remains high, with reported mortality rates exceeding 10% [4].

SD following a low-energy mechanism, such as a fall from standing height, is exceedingly rare, and a comprehensive search of the literature reveals a paucity of documented cases involving such incidents. The purpose of the present study is to present an extremely rare case of SD occurring after a fall from standing height, emphasizing the diagnostic challenges, the importance of maintaining high clinical suspicion even in atypical presentations, and key considerations for successful management.

## Case presentation

A 93-year-old male with a history of hypertension, managed with antihypertensive medication, presented to the emergency department (ED) following a fall from standing height. Upon arrival, he was hemodynamically unstable, exhibiting hypotension (83/54 mmHg), tachycardia (125 beats per minute), and tachypnea (23 breaths per minute). Physical examination revealed pronounced swelling over the right scapular region, axilla, and lateral chest wall, extending around the right shoulder joint. Glasgow Coma Score was 15, with no other clinically evident injuries.

Initially, the fingers and wrist retained normal function, the elbow had limited function, but the shoulder joint was completely nonfunctional. No evident neurological deficits were noted, distal pulses were palpable, and capillary refill time in the right hand was normal. However, within a couple of hours, progressive neurological deficits of the peripheral nerves developed in the right upper limb, raising concern for nerve compression due to an expanding hematoma.

**Figure 1:**
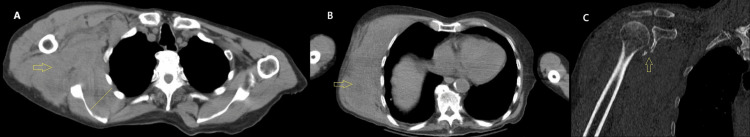
Computed tomography (CT) upon admission. (A) Transverse view showing an excessive hematoma (indicated by the yellow arrow) and lateral displacement of the right scapula (yellow line). (B) Transverse view at a lower level illustrating the right chest wall hematoma (yellow arrow). (C) Coronal view demonstrating an isolated fracture of the right scapular glenoid.

**Figure 2:**
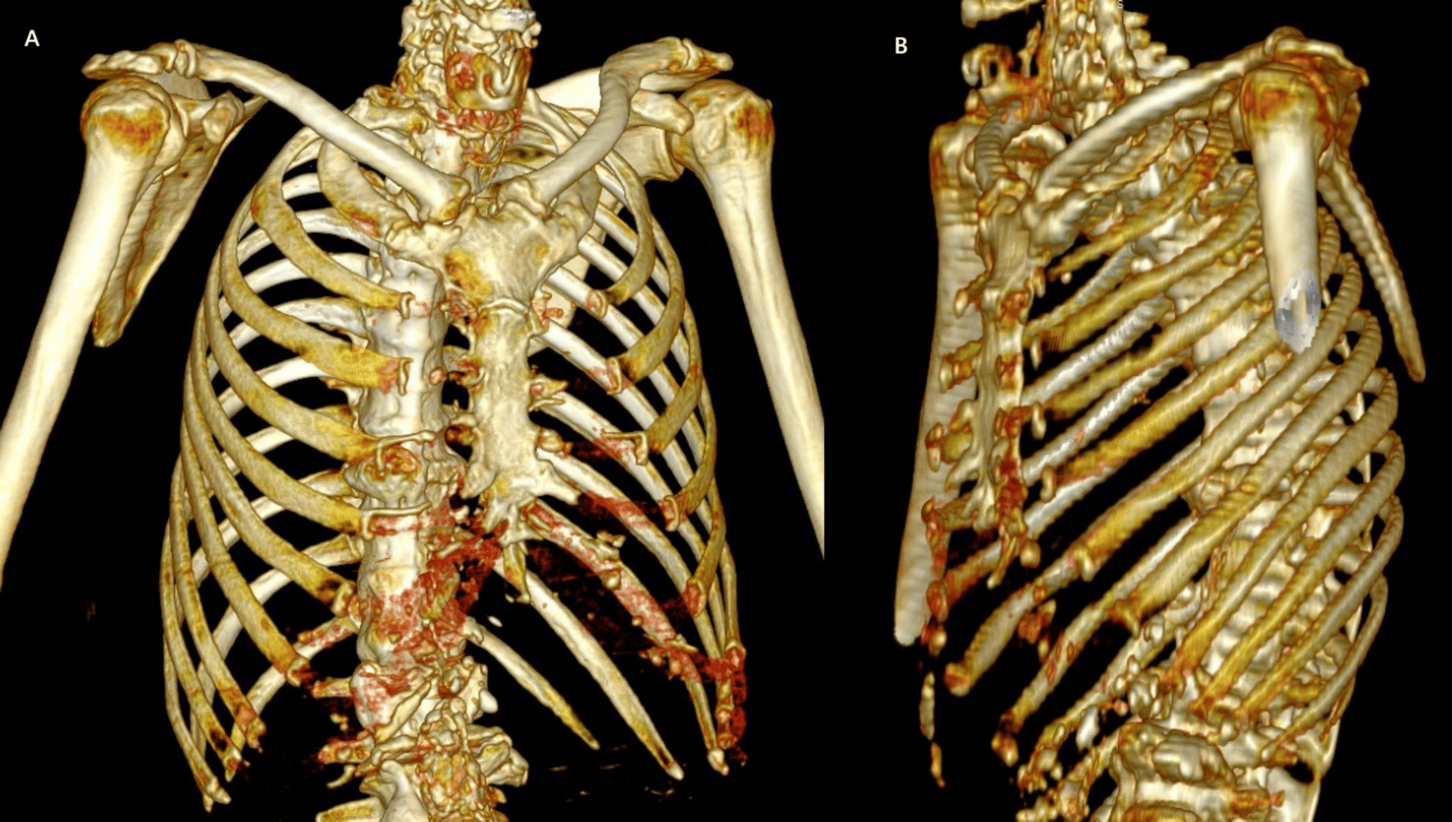
3D reconstruction illustrating the lateral displacement of the right scapula from the thoracic wall (A) compared to the left scapula (B).

A computed tomography (CT) scan was performed, revealing an isolated right scapular glenoid fracture with lateral displacement of the scapula and a large right chest wall hematoma (Figure 1). No rib or clavicular fractures were identified (Figure 2). Despite extensive soft tissue damage, CT angiography ruled out major vascular injury. Given the patient’s age and significant hematoma, conservative management was pursued, with close monitoring. Initial treatment focused on hemodynamic stabilization with targeted fluid resuscitation (lactated Ringer’s solution) and transfusion of four units of packed red blood cells and four units of plasma over 48 hours, considering that the patient developed acute renal failure 24 hours after the trauma.

**Figure 3:**
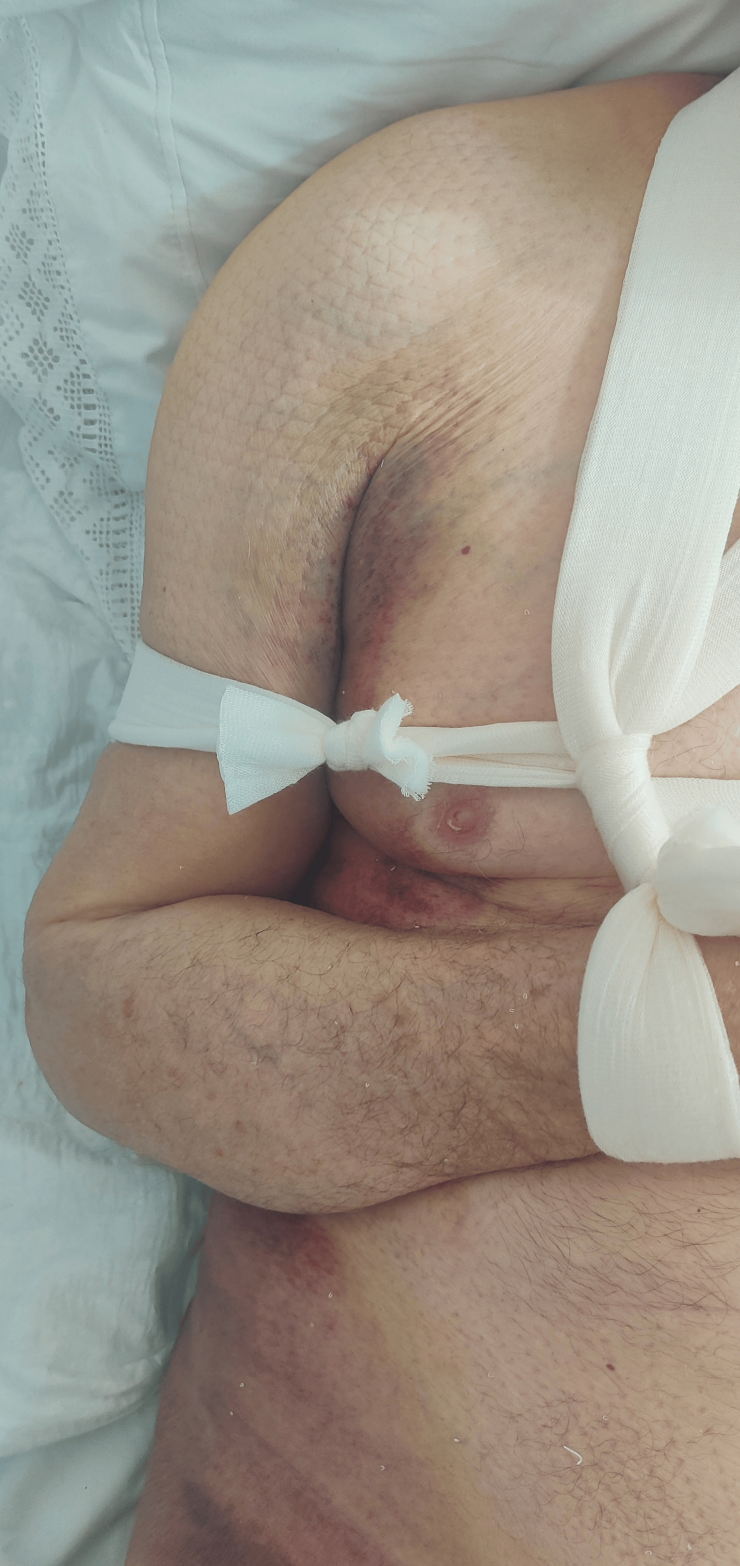
The right upper extremity was immobilized by a Velpeau-type splint.

The right upper limb was initially immobilized by completely wrapping it to the trunk with elastic bandages to minimize dead space and control hemorrhage from the torn musculature. After three days, a Velpeau-type splint was applied for continued immobilization (Figure 3).

**Figure 4:**
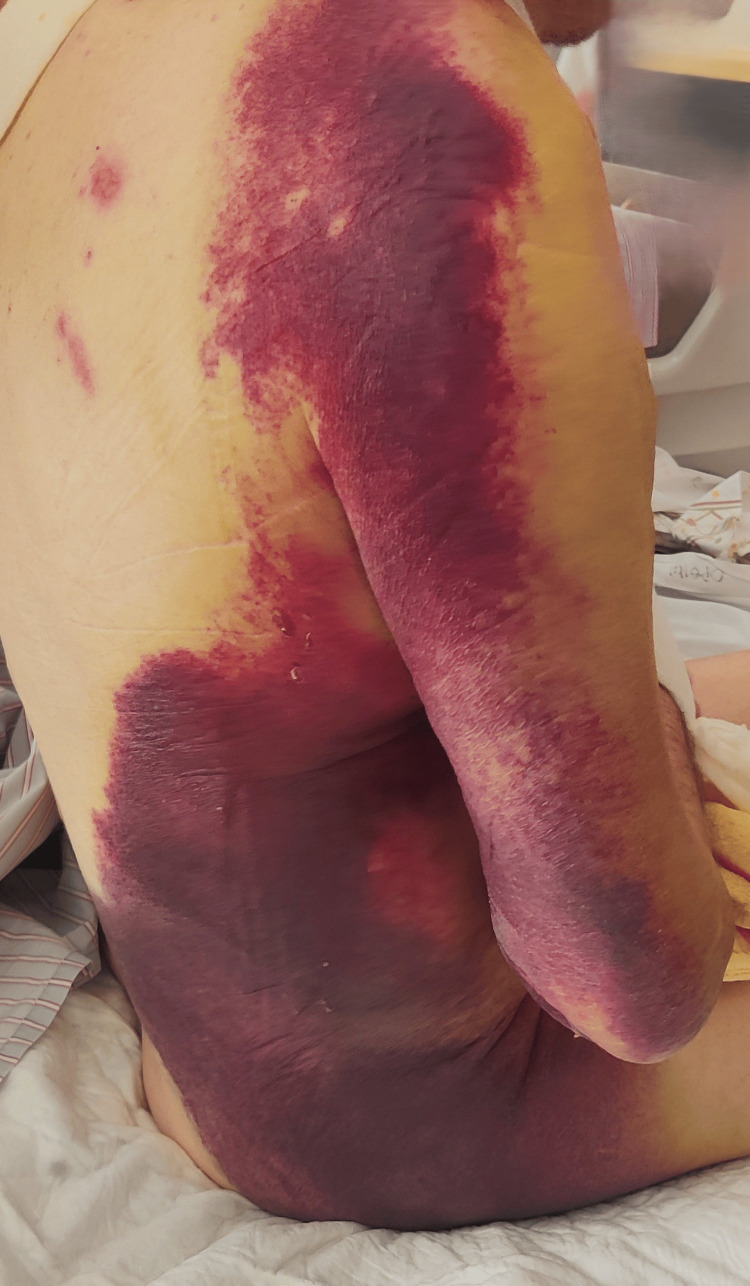
Seven days post-injury: Discoloration of the skin with bruises, as the hematoma expanded inferiorly and extended toward the right upper limb.

**Figure 5:**
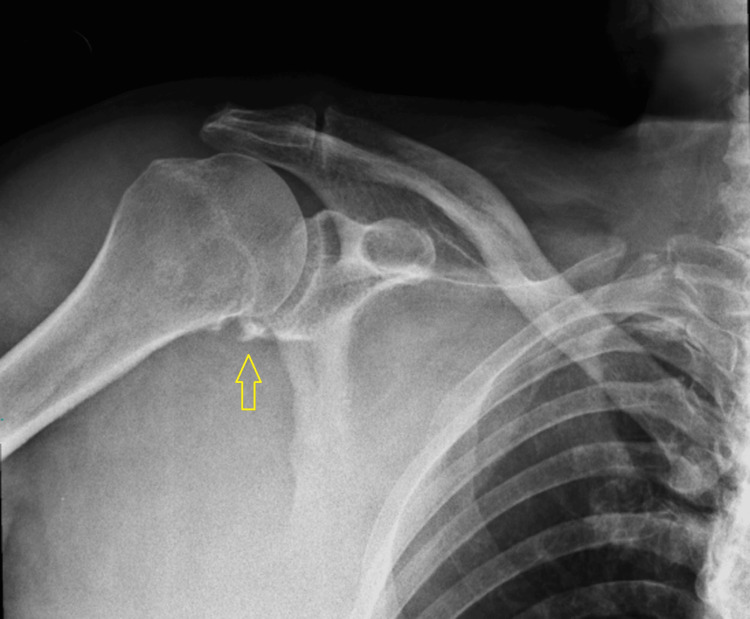
Anteroposterior X-ray of the right shoulder displaying the fracture of the right scapular glenoid (indicated by the yellow arrow).

Over the next few days, the hematoma expanded inferiorly and extended toward the right upper limb but showed gradual resorption without further complications (Figure 4). Neurological function in the affected limb began improving around the fifth day, indicating a degree of spontaneous recovery. Passive range-of-motion exercises for the elbow and wrist were introduced to prevent stiffness. By day 10, neurological function had significantly improved, and the patient was discharged with instructions to continue rehabilitation exercises (Figure 5).

**Figure 6:**
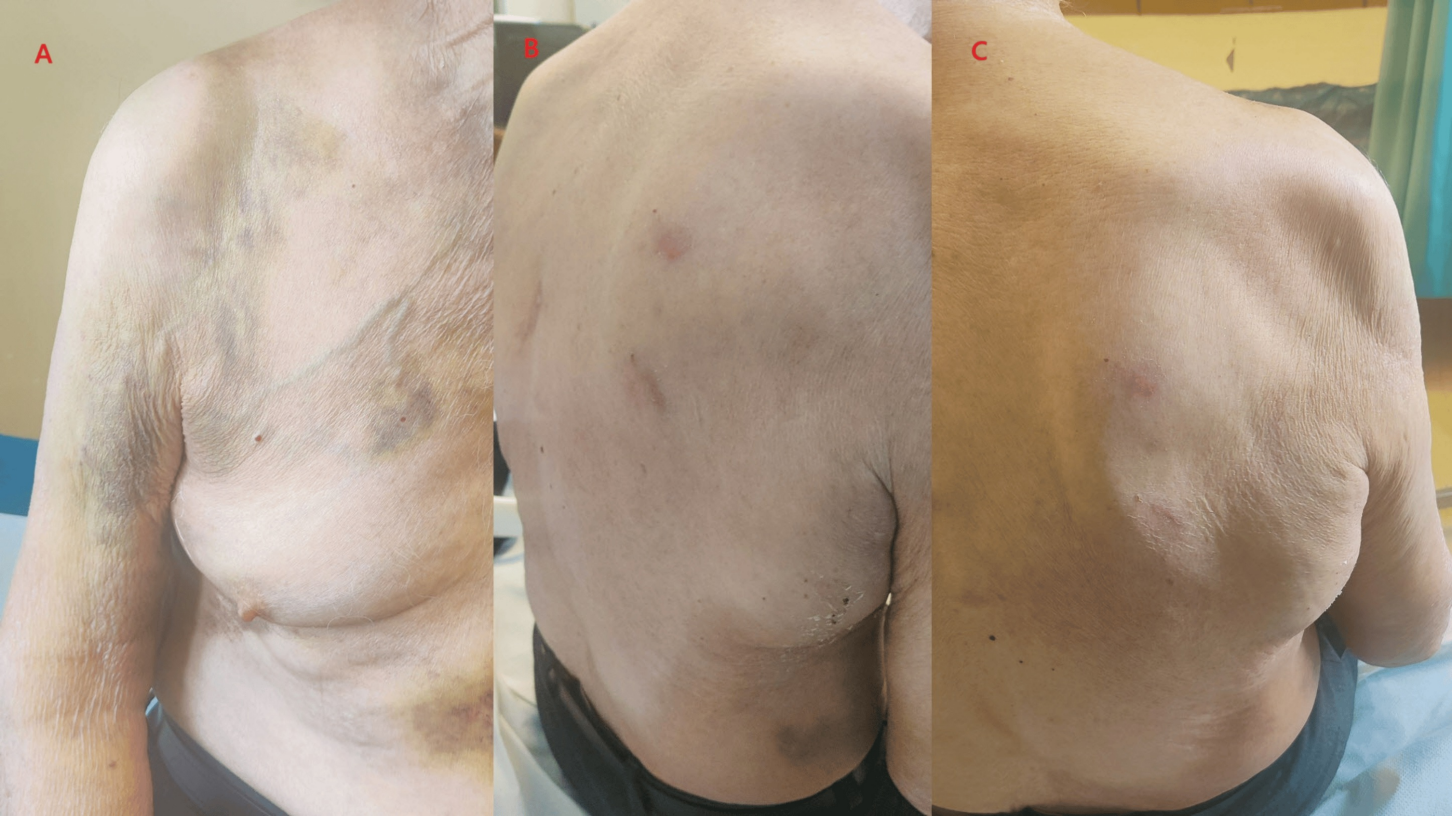
One month post-injury, the hematoma had significantly subsided and the skin had nearly regained its normal color. (A) Anterior view. (B) Posterolateral view. (C) Posterior view.

**Figure 7:**
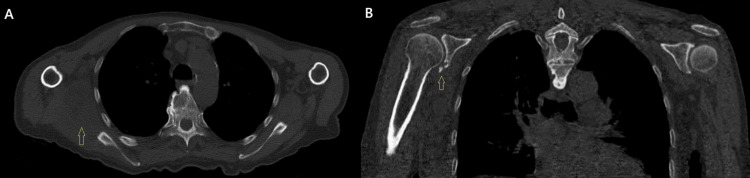
CT one month after the injury. (A) Transverse view depicting the significantly reduced hematoma (indicated by the yellow arrow). The lateral displacement of the right scapula had resolved. (B) Coronal view exhibiting the fracture of the right scapular glenoid.

One month after the trauma, the hematoma had significantly subsided, and the skin had nearly regained its normal color (Figure 6). The Velpeau splint was removed, and a simple sling was provided for periodic use (Figure 7). The neurological function of the upper limb continued to improve. At the two-month follow-up, rehabilitation physiotherapy was initiated to improve muscle strength and proprioception, enhancing the shoulder’s range of motion, while full neurological function had been restored.

By three months post-injury, the patient had resumed independent daily activities, experiencing only mild residual discomfort in the shoulder. Ongoing physiotherapy was recommended to optimize function. At five months, the patient had regained nearly full, painless shoulder range of motion without neurological deficits, demonstrating remarkable recovery given the severity of his initial injury.

## Discussion

SD is traditionally associated with high-energy trauma, where violent forces lead to significant soft tissue damage, osseous injuries, and frequent neurovascular impairment [1-3]. However, our case of a 93-year-old male who sustained SD after a fall from standing height underscores an unusual low-energy mechanism. This presentation, likely influenced by age-related declines in bone quality and soft tissue resilience such as osteoporosis, decreased collagen elasticity and muscle atrophy, demonstrates that even seemingly minor trauma can produce severe injury patterns. Reduced bone density increases fracture risk, while diminished muscle mass and elasticity can lead to decreased shock absorption and joint stability, exacerbating injury severity [5]. To our knowledge, no other similar case has been documented in the literature with such a low-energy mechanism.

SD is primarily caused by strong traction forces on the shoulder girdle, resulting in injuries such as acromioclavicular and sternoclavicular joint disruption, scapular fractures, muscular tears (commonly involving deltoid, pectoralis minor, rhomboid, levator scapulae, latissimus dorsi and trapezius muscles), along with brachial plexus dysfunction [1,3]. The muscles are partially or completely torn first, before any damage occurs to ligaments, blood vessels (mostly subclavian or axillary vessels), or nerves [6].

In our patient, a possible explanation for the mechanism of injury is that as the patient fell, the arm was forcefully abducted or hyperextended, transmitting traction forces through the shoulder girdle. This could have resulted in scapular displacement due to indirect forces. In addition, the rapid deceleration from the fall may have placed excessive strain on the surrounding musculature, leading to partial tearing of stabilizing muscles and eventual scapulothoracic dissociation. The presence of an isolated scapular glenoid fracture with lateral displacement of the scapula, without clavicular or rib fractures, suggests that even low-energy trauma, especially in elderly individuals, can generate sufficient force to disrupt the scapulothoracic articulation. In parallel, the delayed onset of neurological deficits, likely due to hematoma expansion and subsequent nerve compression, highlights the complex, multifactorial nature of these injuries, even when the initial mechanism appears less severe.

Prompt diagnosis is crucial, as SD may initially present insidiously but can progress rapidly. It may also be missed or delayed in polytrauma patients. Common clinical features include severe swelling, palpable hematoma, significant shoulder subluxation, tenderness, and muscle weakness. Despite the serious underlying trauma, the skin often remains intact, masking the extent of the injury [2,7]. A clavicular fracture or acromioclavicular joint disruption is frequently detectable on physical examination [2]. Distinguishing between arterial injury and neurapraxia can be challenging due to overlapping clinical signs. For instance, a patient with complete subclavian artery disruption may experience severe pain and motor or sensory deficits in the affected extremity due to ischemia rather than brachial plexus damage.

Our patient initially presented without a typical clinical manifestation of SD. Aside from swelling over the right scapular region, axilla, and lateral chest wall, and nonfunctionality of the shoulder joint, there were no neurological deficits. Distal pulses were palpable, and the capillary refill time in the affected hand was normal. However, within a few hours, the patient experienced worsening neurological deficits in the right upper limb, likely due to nerve compression. Thus, continuous clinical monitoring is critical, as neurological deterioration can occur over time, emphasizing the need for vigilant reassessment.

To better categorize the diverse clinical presentations of SD and guide diagnostic and therapeutic decision-making, Zelle et al. classified SD into four types, as shown in Table 1 [8]. Based on clinical and imaging findings, our case was classified as Type IIB according to this classification.

Imaging studies play a crucial role in diagnosis. A properly centered, non-rotated chest radiograph can reveal increased soft-tissue density in the shoulder region as well as potential bony or ligamentous injuries [2]. Oreck et al. suggested measuring the distance between the midline thoracic spinous process and the medial border of the scapula on both sides, with a difference greater than 1 cm indicating an SD [2]. Moreover, a scapular index, calculated as the ratio of the distances between the injured and uninjured sides, greater than 1.29 is usually consistent with SD. However, a CT scan is highly recommended upon admission, as it provides a comprehensive image of the affected shoulder girdle along with the chest while simultaneously allowing for an immediate vascular assessment through CT angiography [1,9].

Therefore, in our patient, a CT scan along with CT angiography was performed immediately, bypassing X-rays. The lateral displacement of the scapula and the presence of a large right chest wall hematoma on CT imaging provided crucial diagnostic clues, while CT angiography effectively ruled out major vascular injuries. These findings highlight the necessity of thorough imaging in assessing shoulder girdle trauma, especially when standard radiographs are limited by positioning constraints or overlapping anatomical structures.

Magnetic resonance imaging (MRI) can be utilized to detect cervical root or brachial plexus injuries, as well as related conditions such as pseudomeningocele [1,3]. Upper extremity electromyography (EMG) can assist in identifying affected nerve roots; however, this examination should be conducted no earlier than three weeks post-injury [1,10]. In the present case, neither an MRI, nor an EMG were performed due to the patient's rapid neurological improvement.

SD management should be individualized, prioritizing hemodynamic stabilization while addressing both immediate and long-term functional concerns [1]. Given that SD is often accompanied by other life-threatening injuries, initial management should emphasize polytrauma stabilization, particularly cardiopulmonary resuscitation and hemodynamic support [1]. Once the patient is stabilized, a thorough assessment of the shoulder should determine the appropriate treatment approach. In hemodynamically stable patients, angiography is highly recommended before surgery [1]. In cases of major vascular injury, immediate surgical intervention is necessary to control arterial bleeding as part of the resuscitation protocol. This emergency procedure may involve tamponade packing and rapid suturing to prevent excessive blood loss [1]. After the patient's hemodynamic stabilization, associated fractures should be treated to preserve shoulder girdle integrity. This often involves open reduction and internal fixation, particularly for clavicle fractures, as well as addressing disruptions of the acromioclavicular or sternoclavicular joints [1,3].

In our case, the patient’s initial hemodynamic instability was managed with controlled fluid resuscitation and blood product transfusion. Conservative treatment with immobilization of the affected upper limb tightly wrapped to the trunk, was selected based on the patient’s advanced age, absence of major vascular injury, and evolving neurological symptoms. Notably, despite the initial severity of the injury, the patient demonstrated significant spontaneous neurological recovery. This favorable outcome contrasts with many SD cases, where severe brachial plexus injury or vascular compromise often leads to poor prognosis, frequently requiring surgical intervention or even amputation [4,8,11,12].

## Conclusions

Conclusively, our case demonstrates that while SD is traditionally associated with high-energy trauma, it can also result from seemingly minor mechanisms. Timely recognition, prompt resuscitation, and strategic imaging and management strategies can lead to unexpectedly favorable outcomes, even in severe injuries among elderly patients. Further documentation of such atypical presentations may contribute to refining diagnostic and treatment protocols for this challenging condition.
